# Maternal-fetal-neonatal microbiome and outcomes associated with prematurity

**DOI:** 10.1186/s12887-024-04536-1

**Published:** 2024-02-01

**Authors:** Rita C Silveira, Joseph Y Ting

**Affiliations:** 1 Department of Pediatrics, Hospital de Clínicas de Porto Alegre, Universidade Federal do Rio Grande do Sul, Porto Alegre, Brazil; 2 Division of Neonatal Care, Department of Pediatrics, University of Alberta, Edmonton, AB Canada

## Abstract

Our understanding of the premature gut microbiome has increased rapidly in recent years. However, to advance this important topic we must further explore various aspects of the maternal microbiome, neonatal microbiota, and the opportunities for microbiome modulation. We invite authors to contribute research and clinical papers to the Collection “Maternal-fetal-neonatal microbiome and outcomes associated with prematurity”.

## Main text

New understanding of the gut microbiome and the potential for targeted therapeutic approaches has motivated research around prematurity. However, many issues need to be clarified, especially in this vulnerable population; further research is needed on both the initial determinants of the causes of prematurity and the resulting consequences. Preterm births associated with infection are prevalent, and so a key strategy for preventing prematurity is to prevent the primary infections.

Dynamic changes in microbial diversity in early life are greatly impacted by the intrauterine environment, metabolic and immune regulatory functions that vary according to gestational age, the external environment, postnatal diet, and therapeutic measures such as the use of antimicrobials and probiotics. Internal host characteristics and external factors both influence the establishment of the microbiota [1]. 

There are various challenges that impact the establishment of microbiota in preterm infants, events that can cause alterations in the natural pattern of acquisition, such as type of delivery (C-Section is more common in this population), maternal and neonatal exposure to antibiotics, and environment of the neonatal intensive care unit (NICU). The use of infant milk formula can negatively impact metabolism and the development of the neonatal immune system in comparison with mother’s milk [2]. Differences in preterm infants’ stool microbiota in the neonatal period considering the use of exclusive own mother’s milk and formula in different proportions were found; preterm infants fed with exclusive own mother’s milk presented increased richness and differences in microbial composition from those fed with different proportions of formula [3]. 

Very low birth weight preterm infants who receive exclusively breast milk can rapidly develop their own gut microbiota that is apparently unaffected by the mode of birth or lower gestational age. The definition of a reference profile of the healthy microbiota for preterm infants derives from breast milk, the ideal source of nutrition for these babies.

It is of note that a study found maternal vaginal microbiota had low similarity with the initial colonization of the baby’s intestine, and maternal vaginal clusters dominated by Lactobacillus were not associated with Lactobacillus in the babies’ meconium at birth [4]. 

It is known that increases in microbial species and functions mature the gut microbiome, increasing its richness and diversity [5]. Immunological immaturity is observed in germ-free experimental models, and humans living on farms have greater functional microbial diversity and less susceptibility to chronic inflammatory diseases than urban dwellers. Lifestyle modernization can cause the progressive loss of the most flexible and responsive microbes to environmental exposures. Therefore, early modulation of the microbiome, since pregnancy and peripartum, with precise nutrition and rational microbial supplementation, can promote or restore functional microbial networks impaired by many conditions associated with prematurity [6]. Human milk oligosaccharides (HMOs) and the breast milk microbiome are immunomodulators, providing intestinal homeostasis through regulation of the microbiome and protection of the intestinal barrier [7]. 

Several studies have shown that preterm infants who develop necrotizing enterocolitis (NEC) present early gut microbiome dysbiosis; lower microbial diversity and an abnormal succession of the microbial community before NEC diagnosis mainly due to organ immaturity, antibiotic use, and hospital microbial environment. The reduction in microbial diversity contributes to the development of diseases. Enteral probiotic supplements, HMOs, probiotics, and symbiotic combinations in protecting against necrotizing enterocolitis have been studied [4]. 

The NICU is a specialized clinical environment that caters to the needs of delicate newborns who are highly susceptible to severe infections. The usual clinical approach involves early and preemptive treatment with potent antimicrobials [8, 9]. However, substantial differences in the rates of antimicrobial use were noted across various NICUs due to the absence of widely accepted evidence-based guidelines on the type and duration of antimicrobials to use for common conditions [9, 10]. Retrospective studies have demonstrated that antimicrobials are frequently prescribed and continued in clinical situations where no clear benefit has been demonstrated [11]. 

The inappropriate use of antimicrobials can cause gut dysbiosis and the emergence of antibiotic-resistant organisms. Higher rates of antibiotic utilization in preterm infants, without confirmed sepsis or NEC have been associated with increased mortality or major morbidity, as well as a composite outcome of death or adverse early neurodevelopmental outcomes [12]. The disturbance in microbiomes has been hypothesized as one of the major causes, leading to the hotly debated terms “gut-lung axis” and “gut-brain axis” [13, 14]. 

Studies have also indicated that preterm birth is linked to an increased risk of a wide range of long-term consequences that may persist into adulthood. Further research is required to understand the extent to which the aforementioned adverse health outcomes are attributed to disturbed microbiomes and whether they are potentially amenable to early-life intervention.

We hope that this collection will provide a platform for exploring three major research topics across this field: firstly, the role of the maternal microbiome in the context of prematurity; secondly, the neurological outcomes associated with neonatal microbiota, including the brain-gut axis; and finally, the opportunities for microbiome modulation relating to prematurity outcomes.

## Data Availability

Not applicable.

## Abbreviations

NICU: neonatal intensive care unit
NEC: necrotizing enterocolitis
HMOs: Human milk oligosaccharides
